# Pathogen distribution and bacterial resistance in children with severe pneumonia: A single-center retrospective study

**DOI:** 10.1097/MD.0000000000027128

**Published:** 2021-09-03

**Authors:** De-Quan Su, Hong-Lin Huang, Zhi-Qiang Zhuo

**Affiliations:** Department of Pediatric Internal, Children's Hospital of Fudan University Xiamen Branch, Xiamen, China.

**Keywords:** antibiotic resistance, bacterial cultivation, pediatrics, severe pneumonia

## Abstract

To examine the etiological distribution of pathogens in pediatric patients with severe pneumonia and analyze the drug resistance of major pathogen species.

Nasopharyngeal secretion specimens were collected for bacterial culture from pediatric patients admitted to the Xiamen children's hospital who were diagnosed with severe pneumonia from January 2016 to December 2019. Pathogen species were detected by quantitative polymerase chain reaction, direct immunofluorescence, and bacterial culture and we examined the drug susceptibility of the bacterial pathogens.

At least 1 species of the pathogen was detected in 576 of 734 patients and a total of 444 bacterial samples were isolated, of which 284 were gram-negative and 160 were gram-positive. The most frequently detected bacteria were *Haemophilus influenzae*, *Streptococcus pneumonia*, *Staphylococcus aureus*, *Klebsiella pneumoniae*, and *Escherichia coli*. In addition, we isolated 186 viral samples, of which the majority were respiratory syncytial virus (n = 90) and adenovirus (n = 70) as well as 142 *Mycoplasma pneumonia* samples.

Gram-negative bacteria are dominant among the pathogens causing severe pneumonia in pediatric patients and the major pathogen species are resistant to a variety of antibiotics. Appropriate antibiotic use has an important role in preventing the emergence of resistant strains.

## 1 Introduction

Pneumonia is a common and frequently occurring disease among children, especially infants. According to the data, pneumonia accounted for 12.2% of deaths among children under 5 years old in 2015.^[1]^ Pneumonia in children is characterized by complex disease progression, multiple types of pathogenic bacteria, and strong pathogenicity. It is relatively difficult to treat^[2]^ and 7% to 13% develop into severe pneumonia, which may lead to multi-organ damage and dysfunction.^[3]^ Without timely treatment, children may continue to deteriorate and succumb to the disease. Early and accurate etiological detection and selection of proper antibiotics can significantly improve the prognosis. With the extensive use of antibiotics and the occurrence of resistant strains in recent years, the composition of pathogenic bacteria and drug susceptibility of severe pneumonia have already undergone great changes in different areas. Therefore, examining the epidemic characteristics of the pathogens and their drug resistance will be critical for selecting treatment strategies and improving the success rate. In this study, we detected pathogens of hospitalized children with severe pneumonia using conventional bacterial culture, quantitative polymerase chain reaction, and direct immunofluorescence. Furthermore, we characterized the etiology and bacterial resistance to antibiotic agents.

## 2 Materials and methods

### 2.1 Study subjects

We enrolled a total of 734 pediatric patients with severe pneumonia admitted to the Xiamen children's hospital from January 2016 to December 2019. All study subjects met the diagnostic criteria for severe pneumonia. Severe pneumonia was defined as the presence of lower chest indrawing (definite inward movement of the lower chest during quiet breathing) and/or with general danger signs (not able to drink, persistent vomiting, convulsions, lethargy or unconscious, stridor in the calm child or severe malnutrition), in children presenting with cough or difficult breathing.^[3]^ The cohort consisted of 462 males and 272 females, of whom 378 patients were less than 1 year old, 188 patients were 1 to 3 years old, 138 patients were 3 to 6 years old, and 30 patients were 6 to 14 years old. This study was conducted in accordance with the 1964 declaration of Helsinki and its later amendments and was approved by the Ethics Committee of the Children's Hospital of Fudan University Xiamen Branch. Written informed consent was obtained from all participants’ parents or legal guardians.

### 2.2 Sample acquisition and processing

Within 24 hours after hospitalization, the patients were given normal saline to clean their mouths. The sputum was obtained from the deep airways by negative pressure with a disposable suction catheter via the nose. For patients requiring mechanical ventilation, sputum was obtained using a tracheal catheter. Sputum quality was considered adequate and it contained ≤10 epithelial cells and ≥25 leukocytes under low magnification.

Direct immunofluorescence kits (Chemieon, USA) was used according to the manufacturer's recommendations to detect the following respiratory viruses: respiratory syncytial virus (RSV), adenovirus, influenza virus A, influenza virus B, parainfluenza virus 1, parainfluenza virus 2, and parainfluenza virus 3. *Mycoplasma pneumoniae* was detected by quantitative polymerase chain reaction and MP serum IgM antibody was detected using particle agglutination assay. Gene copy ≥5 × 10^2^/mL or IgM titres ≥1:160 indicated infection. Phoenix-100 automated identification and susceptibility testing system (BD, USA) was used to culture and identification of bacterial samples and drug sensitivity analysis.

### 2.3 Statistical analysis

The statistical analysis was performed using SPSS 21.0. Counting data are presented as percentages and non-normally distributed data are displayed as median. *χ*^2^ test or Fisher exact test were used for statistical comparison. *P* < .05 was considered statistically significant.

## 3 Results

Among 734 enrolled pediatric patients with severe pneumonia, we detected at least 1 pathogen in 576 patients while no pathogen was present in the remaining 178 patients, showing a detection rate of 78.47%. Four hundred forty four cases of bacteria were detected (56.2%), 186 cases of viruses were detected (23.54%), 142 cases of *M pneumoniae* were detected (17.97), and 18 cases of fungi were detected (2.28%).

A total of 444 (56.20%) bacterial samples were isolated, of which 284 (63.96%) were gram-negative and 160 (36.04%) were gram-positive. The top 3 bacterial detected included *Haemophilus influenzae* (12.91%), *Streptococcus pneumoniae* (10.38%), and *Staphylococcus aureus* (7.09%). The top 3 viruses detected included respiratory syncytial virus (11.39%), adenovirus (8.86%), and parainfluenza virus 3 (1.52%). And 142 cases (17.97%) of mycoplasma were detected (Table 1). There were significant differences in the distribution of bacterial, viral, MP, and fungal infections between different age groups (*P* < .001) (Table 2).

**Table 1:** Etiology of pathogens in paediatric patients with severe pneumonia.

Pathogen	Positive (n)	Proportion (%)
Bacteria	444	56.20
*Haemophilus influenzae*	102	12.91
*Streptococcus pneumoniae*	82	10.38
*Staphylococcus aureus*	56	7.09
*Klebsiella pneumoniae*	50	6.33
*Escherichia coli*	38	4.81
*Pseudomonas aeruginosa*	32	4.05
*Moraxella catarrhalis*	28	3.54
*Haemolytic staphylococcus*	22	2.78
*Acinetobacter baumannii*	14	1.77
*Bordetella pertussis*	10	1.27
*Stenotrophomonas maltophilia*	6	0.76
*Enterobacter cloacae*	2	0.25
Elizabethan meningeal septicaemia	2	0.25
Respiratory virus	186	23.54
Respiratory syncytial virus	90	11.39
Adenovirus	70	8.86
Parainfluenza virus 3	12	1.52
Influenza virus A	6	0.76
Influenza virus B	4	0.51
Parainfluenza virus 1	2	0.25
Parainfluenza virus 2	2	0.25
Mycoplasma	142	17.97
Fungi	18	2.28

**Table 2:** Bacterial and viral infections by age.

Age	Bacteria (n)	Virus (n)	MP	Fungal
1–12 months	232	106	10	11
1–3 years	126	48	22	2
3–6 years	66	26	46	4
6–14 years	20	6	64	1
*χ* ^2^	131.92	64.02	121.91	32.1
*P*	<.001	<.001	<.001	<.001

Among the main gram-negative bacteria, *H influenzae* has a resistance rate of more than 75% to compound trimethoprim and ampicillin, but a sensitivity rate of 100% to cefotaxime, levofloxacin, ceftriaxone, meropenem, ampicillin/sulbactam, and cefoperazone/sulbactam; *Escherichia coli* and *Klebsiella pneumoniae*, the resistance rate to ampicillin, cefotaxime, ceftriaxone, and cefpod is more than 50%, but the sensitivity rate to carbapenem antibiotics (meropenem, ertapenem, and imipenem) is 90%, and the sensitivity rate to β-lactamase inhibitor compound preparations (except ampicillin/sulbactam) is above 80% (Table 3).

**Table 3:** Drug resistance of major gram-negative bacteria to antibiotic agents.

Antibiotic agent	*Haemophilus influenzae*, n = 51 (%)	*Klebsiella pneumoniae*, n = 25 (%)	*Escherichia coli*, n = 19 (%)
Ampicillin	78 (76.47)	50 (100.00)	26 (68.42)
Sulfamethoxazole	90 (88.24)	24 (48.00)	20 (52.63)
Levofloxacin	0	6 (12.00)	16 (42.10)
Cefoxitin	32 (31.37)	8 (16.00)	6 (15.79)
Cefotaxime	0	30 (60.00)	26 (68.42)
Ceftriaxone	0	28 (56.00)	26 (68.42)
Meropenem	0	0	0
Ampicillin/sulbactam	0	28 (56.00)	26 (68.42)
Cefoperazone/sulbactam	0	0	0
Gentamicin	22 (21.57)	32 (64.00)	26 (68.42)

Among the main gram-positive bacteria, *S pneumoniae* is resistant to erythromycin, tetracycline, and SMZ as high as 85%; *S aureus* is resistant to penicillin and erythromycin as high as 50%; and the 2 sensitivity rates to levofloxacin, vancomycin, linezolid, and meropenem is 100% (Table 4).

**Table 4:** Drug resistance of major gram-positive bacteria to antibiotic agents.

Antibiotic agent	*Streptococcus pneumoniae*, n = 41 (%)	*Staphylococcus aureus*, n = 28 (%)
Erythromycin	82 (100.00)	30 (53.57)
Sulfamethoxazole	72 (87.80)	10 (17.86)
Tetracycline	70 (85.37)	20 (35.71)
Penicillin	16 (18.51)	42 (75.00)
Ceftriaxone	12 (14.63)	8 (14.29)
Cefotaxime	12 (14.63)	8 (14.29)
Benzoxacillin	24 (29.27)	14 (25.00)
Levofloxacin	0	0
Vancomycin	0	0
Linezolid	0	0
Meropenem	0	0

## 4 Discussion

In this study, we found higher bacterial infection rates compared with virus infection rates in children with severe pneumonia across all age groups. The detected bacteria species were mainly gram-negative bacteria and were consistent with previous reports,^[4,5,6,7]^ although other studies have described different pathogen compositions,^[8,9]^ suggesting that the bacterial spectrum causing pneumonia may take change across different regions and disease states. A clear understanding of the local bacterial spectrum that causes severe pneumonia would guide clinicians to select appropriate antibiotics and improve the success rate for pediatric patients with severe pneumonia.

The main bacteria detected in our cohort was *H influenzae* and we observed high resistance to sulfamethoxazole and ampicillin, although it was susceptible to cefotaxime and ceftriaxone. Ampicillin is the historical drug of choice for treating *H influenzae* infection, but in recent years, ampicillin resistance has increased substantially from 19% to 29.1% in Europe and the United States^[10,11]^ and even as high as 63.5% to 69.4% in Japan and South Korea,^[12]^ suggesting that it should no longer be the first-line treatment for *H influenzae*. Furthermore, resistance to sulbactam, amoxicillin, and clavulanic acid is also relatively high.^[13]^ Thus, ceftriaxone and cefotaxime may be better primary treatment options. The second most common bacteria were *S pneumoniae* and we observed high resistance to erythromycin, tetracycline, and sulfamethoxazole with lower resistance to penicillin, cefotaxime, and ceftriaxone. However, it was extremely sensitive to vancomycin and linezolid. These data are consistent as reported previously^[13,14]^ and the high resistance to multiple antibiotics may be related to the improper use of macrolide antibiotics. Another explanation could be the phenomenon of cross-resistance as demonstrated by Yu et al^[15]^ However, penicillin and second-generation and third-generation cephalosporins are still effective to combat most *S pneumoniae* isolates. Compared with previous literature,^[16]^ the presence of drug-resistant pathogens in our study was decreased and may be related to the strict control of antibiotic use and management of antibiotics in China.^[17]^

The most common extended-spectrum beta-lactamase-producing bacteria were *E coli* and *K pneumonia*. Over half of the *E coli* and *K pneumonia* isolates were resistant to ceftriaxone, ampicillin, and ampicillin/sulbactam in our study, but were susceptible to piperacillin/tazobactam and carbapenems. Furthermore, we found that *E coli* and *K pneumonia* showed different degrees of resistance to common antibiotics like penicillin and cephalosporins with mild resistance to carbapenems and quinolones, which was similar to previous studies.^[18–20]^ Taken together, these results indicate that piperacillin/tazobactam may be selected for treating *E coli* and *K pneumonia* infection while carbapenems may be used for drug-resistant patients.

Research has shown that RSV is an important cause for the development of severe pain. The detectionumonia in children^[21]^ rate of viruses in children with severe pneumonia in Chongqing was as high as 72.3% and was dominated by RSV.^[22]^ In our study, the detection rate of RSV ranked highest among the detected respiratory viruses and was consistent with previous reports from Suzhou,^[22]^ indicating that RSV is an important cause of severe pneumonia in children in Xiamen.

There are sporadic infection cases of *M pneumoniae* throughout the year across different regions with seasonal incidence patterns. Our data indicate that the incidence in autumn was the highest and was lower in summer with the lowest rates in winter and spring, which was inconsistent with the patterns reported in Beijing.^[23]^ Certain temperatures and humidity levels, especially rainfall amount, are closely related to the spread of *M pneumonia*^[24]^ and maybe the reason for high incidence during autumn in Xiamen.

In conclusion, severe pneumonia in our study was mainly found in the younger age group and was mainly due to bacterial infections. Gram-negative bacteria were the main *H influenzae* and gram-positive bacteria were primarily composed of *S pneumonia* and *S aureus*. RSV was an important pathogenic virus and *M pneumoniae* was mostly seen in pre-school children.^[25–27]^ As pathogen culture requires a certain period of time^[28]^ and the initial treatment of pneumonia patients is dominated by empirical treatment, clinicians should reinforce monitoring of drug resistance in different regional pathogens. It is critical to use the proper antibiotics and follow the treatment standards to prevent further increasing drug resistance in bacteria.

## Acknowledgments

We thank the participants of the study.

## Author contributions

**Conceptualization:** De-Quan Su.

**Data curation:** De-Quan Su, Hong-Lin Huang.

**Formal analysis:** De-Quan Su, Hong-Lin Huang.

**Funding acquisition:** De-Quan Su.

**Project administration:** Zhi-Qiang Zhuo.

**Resources:** De-Quan Su.

**Software:** Hong-Lin Huang.

**Visualization:** Hong-Lin Huang.

**Writing – original draft:** De-Quan Su.

**Writing – review & editing:** Hong-Lin Huang, Zhi-Qiang Zhuo.
